# Silent Damage of Noise on Cochlear Afferent Innervation in Guinea Pigs and the Impact on Temporal Processing

**DOI:** 10.1371/journal.pone.0049550

**Published:** 2012-11-21

**Authors:** Lijie Liu, Hui Wang, Lijuan Shi, Awad Almuklass, Tingting He, Steve Aiken, Manohar Bance, Shankai Yin, Jian Wang

**Affiliations:** 1 Department of Physiology and Pharmacology, Medical College of Southeast University, Nanjing, China; 2 Department of Otolaryngology, 6^th^ Affiliated Hospital, Jiaotong University, Shanghai, China; 3 Department of Physiology & Biophysics, Dalhousie University, Halifax, Canada; 4 School of Human Communication Disorders, Dalhousie University, Halifax, Canada; 5 Division of Otolaryngology, Head and Neck Surgery, Department of Surgery, Dalhousie University, Halifax, Canada; Emory Univ. School of Medicine, United States of America

## Abstract

Noise-exposure at levels low enough to avoid a permanent threshold shift has been found to cause a massive, delayed degeneration of spiral ganglion neurons (SGNs) in mouse cochleae. Damage to the afferent innervation was initiated by a loss of synaptic ribbons, which is largely irreversible in mice. A similar delayed loss of SGNs has been found in guinea pig cochleae, but at a reduced level, suggesting a cross-species difference in SGN sensitivity to noise. Ribbon synapse damage occurs “silently” in that it does not affect hearing thresholds as conventionally measured, and the functional consequence of this damage is not clear. In the present study, we further explored the effect of noise on cochlear afferent innervation in guinea pigs by focusing on the dynamic changes in ribbon counts over time, and resultant changes in temporal processing. It was found that (1) contrary to reports in mice, the initial loss of ribbons largely recovered within a month after the noise exposure, although a significant amount of residual damage existed; (2) while the response threshold fully recovered in a month, the temporal processing continued to be deteriorated during this period.

## Introduction

Noise exposure is the most common cause of sensorineural hearing loss (SNHL), which is one of the most common neurological disorders [1] in modern society. Currently, the effects of noise on hearing are mainly evaluated with tests addressing auditory sensitivity (hearing thresholds), as are safety standards for industrial and environmental noise control. However, a recent study in mice revealed that exposure to noise at levels that do not cause permanent hearing loss (i.e., permanent threshold shift; PTS), may still cause massive damage to the synapses between cochlear inner hair cells (IHCs) and type I spiral ganglion neurons (SGNs), followed by a slowly developing process of degenerative SGN death [2]. Similar noise exposure in guinea pigs was found to cause a similar delayed SGN death process, but on a much smaller scale [3]. This SGN damage is “silent” in that it will not be detected by standard tests of auditory threshold. These reports suggest that “silent” SGN death is likely a common phenomenon in mammalian ears, but that quantitatively large cross-species differences may exist. This cross-species variation must be studied before extrapolation to humans is possible.

Synapses between IHCs and SGNs are characterized by the presence of a large presynaptic organelle, the synaptic ribbon [4]. In the studies mentioned above, noise-induced SGN degeneration was preceded by a loss of synaptic ribbons in IHCs [2]. This ribbon loss was considered to be an indicator of synaptic damage. In the mouse study, the initial ribbon loss was approximately 60%. The ribbon count recovered by only 10% one week after the noise, resulting in a permanent 50% loss that did not change further. It is interesting to note that this 50% permanent loss of ribbons was matched by the percentage SGN loss observed 2 years after the noise exposure [2]. The ribbon synapses of these SGN neurons onto IHCs are likely not re-established, resulting in a loss of trophic support to the SGNs from IHCs and supporting cells, and causing degenerative death. The concordance between the quantity of ribbon loss and quantity of the delayed SGN loss suggests that the ribbon count is a good indicator for the quantity of functional synapses since the survived SGNs are likely to have intact synapses with IHCs which appears to be correctly indicated by the number of ribbons.

In a similar study in guinea pigs from Lieberman’s group, a similar amount of ribbon loss (50–60%) was found 10 days after noise, however the corresponding SGN loss measured 2 years later was much smaller [3]. This discrepancy suggests that compared to mice, more ribbon synapses in the guinea pig cochleae might have been repaired. This is consistent with older studies in which noise induced IHC-SGN synapse damage was found to be largely reversible, as summarized by Puel [5], although these studies have been criticized by Liberman’s group as not being quantitative and not being conducted over a long enough period [2]–[3]. However, while the ribbon count may be a good indicator for synaptic damage and repair, no quantitative data are available for the recovery of ribbons after their initial loss in the cochlea of guinea pigs.

Although a small proportion of conventional synapses may co-exist [6], the afferent synapse between IHCs and SGNs are mainly of the typical ribbon type [4], [7]–[11], which is capable of high-speed neurotransmitter release in response to graded changes of membrane potential and ongoing recycling of released neurotransmitters. Because of these properties, ribbon synapses are recognized to play a critical role in the temporal signal processing in the cochlea [4], [7]–[8], [12]. Massive damage to IHC-SGN ribbon synapses likely compromises the temporal resolving power of the auditory system, so impaired temporal processing may be an important marker for noise-induced damage in the absence of threshold change. However, information about the consequences of noise-induced ribbon synapse damage for auditory temporal resolution is not available.

The present study combined physiological examination with immunohistological evaluation to revisit the effects of noise exposure on cochlear function and morphology in guinea pigs. There were two main objectives: (1) to quantify ribbon synapse damage and recovery over time (i.e. dynamically) and the potential loss of SGNs; (2) to determine whether damage to ribbon synapses impacts auditory temporal processing.

## Materials and Methods

### A. Animals and General Experimental Protocol

In total, 60 male adult (2–3 months old) guinea pigs (Albino) were subjects (Charles River Co. Canada and Shanghai Sheng-Wang Co. Limited, Shanghai) in this experiment. They all passed Preyer reflex testing and an otoscopic exam. Their body weights were between 300 and 350 g at experiment onset. In all animals, hearing status was verified using auditory brainstem responses (ABRs) and those with abnormal hearing were not used. All animal procedures were approved by the University Committee of Laboratory Animals of Shanghai Jiao Tong University, China (Permit Number: SYXK(H2011-0128) and Dalhousie University, Canada (Permit Number:10-025).

The study was divided into two parts. In the first part, 30 animals were used to evaluate SGN damage after a brief exposure to 110 dB SPL broadband noise for 2 h. They were randomly assigned to the experimental group (n = 24) or the control group (n = 6). The control group established our norms for compound action potential (CAP) and counts of SGNs and auditory nerve fibers (ANFs) (12 cochleae from 6 animals). The 24 animals in the active experimental group were divided into four subgroups based on the time they were sacrificed for morphological analysis: 1 day (d), 1 week (w), 1 month (m) or 6 m post noise exposure (PE). The sample size was 6 in each subgroup. After hearing function was evaluated by recording ABRs and CAPs, one ear in each animal was used for ANFs/SGNs quantification and the other ear was used to examine the loss of hair cells. However, for the 6mPE group, both ears were used for the ANF/SGN counts. ABR threshold, CAP amplitude and morphological measures from the noise-exposed animals were compared with those of the controls.

In the second part of the experiment, potential deterioration in synaptic ribbons and temporal processing was investigated after a brief exposure to 105 dB SPL broadband noise for 2 h. For this section, 30 guinea pigs were employed, which were randomly assigned to the four experimental groups (n = 24, 6 each for 1dPE, 1wPE, 2wPE and 1mPE), and the control group (n = 6) in which norms were established for corresponding auditory function and morphology.

### B. Noise Exposure

**Figure 1:**
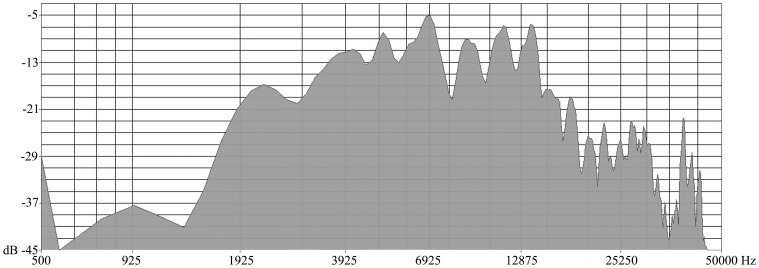
The power spectrum of noise employed in this study.

The animals in the noise groups were exposed to a single dose of broadband noise at either 105 or 110 dB SPL for 2 h. During the exposure, the animal was awake and unrestrained in a small cage 60 cm below the horn of the sound-delivery loudspeaker (TW67) in a sound booth. The electrical Gaussian noise was generated by a System III processor from Tucker-Davis-Technologies (TDT, FL, USA) and delivered to the speaker after power amplification. The acoustic spectrum of the sound is shown in (Figure 1) and mainly distributed between 1.5 and 20 kHz due to the frequency response of the speaker. The noise was monitored using a 1/4 inch microphone linked to a sound level meter (Larson Davis 824). The variation of the sound level across the cage was less than 1 dB.

### C. Physiological Tests

For ABR and CAP recordings, the animal was anesthetized with ketamine+Xylacine (40 mg/kg +10 mg/kg, respectively, i.p.) and the body temperature maintained at 38°C with a thermostatic heating pad. Three subdermal needle electrodes were used to record ABRs. To record the CAPs, a silver ball electrode was placed on the round window membrane via a surgery opening the mastoid. The electrode was fixed in place with dental cement.

TDT hardware and software (BioSig) was used for stimulus generation and bio-signal acquisition. Acoustic stimuli used in our study were: 1) tone bursts of 10-ms duration with cos^2^ gating, 0.5-ms rise/fall time; and 2) equal-level paired clicks of 80 µs duration with inter-click intervals (ICI) varied from 20 ms to 1 ms. Stimuli were played through a speaker (MF1 or FT28) which was placed 10-cm in front of the animal’s head. Evoked responses were sampled at 25 kHz and preamplified by a TDT RA16PA with a gain of 20 and averaged 1000 times for ABR and 100 times for CAP.

For measuring hearing threshold, the ABR was recorded with tone bursts presented at a rate of 21.1/sec at frequencies from 1 to 32 kHz in octave steps. At each frequency, threshold was determined as the lowest level at which a repeatable wave III response could be obtained, in 5 dB steps descending from 90 dB SPL. To evaluate auditory temporal processing ability, the ABR was recorded using paired-clicks with inter-click intervals ranging between 1 to 20 ms and an overall repetition rate of 11.1/sec, presented at a suprathreshold level (70 dB peSPL (peak equivalent sound pressure level)). Root-mean-square (RMS) amplitude was calculated for the response evoked by the second click, labeled ABR2 in the present report. The calculation of the ABR2 RMS amplitude was not referred to any specific wave peak but was performed in a 4-ms window, starting at 1.5 ms after the onset of second click (see results). We used the RMS instead of the measurement of a specific wave peak to avoid uncertainty in exact peak labelling and measurement. When the ICI was smaller than 4 ms, the response to the second click likely overlapped with that to the first click. In such cases (for ICI ≤ 4 ms), we performed subtraction from the ABR recorded at 20 ms ICI to eliminate the response to the first click before calculating the RMS amplitude for the response to the second click. For understanding how the RMS amplitude changed as a function of ICI, an ABR2 RMS amplitude ratio was calculated as ABR2 amplitude versus the maximal ABR2, which usually occurred at the largest ICIs (20 or 10 ms). Both absolute ABR2 amplitude and the normalized ABR2 amplitude declined as the ICI reduced and a quicker decline of ABR2 with ICI was considered to indicate poorer temporal processing. These functions were compared across animals tested at different times post-noise-exposure to show the impact of noise on auditory temporal processing.

The CAP was also recorded with tone bursts presented at rate of 21.1/sec. The amplitude between the first negative depression and subsequent positive wave was measured to evaluate the impact of noise exposure on CAP input/output (I/O) functions.

### D. Morphology

After functional testing, the animal was sacrificed and its cochlea was used for immunostaining and quantification of ANFs and SGNs or IHC-SGN synaptic ribbons. To count ANFs and SGNs, we used our previously published protocols [13]. Briefly, the cochlea was perfused with 2% glutaraldehyde in PBS buffer and then immersed in the fixative for 6 hours at 4°C, followed by decalcification in 5% EDTA. After further fixation in 1% osmium acid for one hour at room temperature and dehydration in grade ethanol, the sample was embedded with Epon using standard procedures. Semi-thin cross-sections of 1.5–2 µm was made, transferred to a glass slide and stained with 1% Toluidine blue for 1 minute, and then examined under a light microscope. The number of SGN cell bodies was counted in Rosenthal’s canal at two-sides of the modiolus in each turn. At each location, 10 sections were taken to cover a distance over 0.2 mm and the SGN cell body counts were averaged across the 10 sections. The number of auditory nerve fibers (dendrites from the SGN) was counted in the sections crossing the habenular perforatae. For each ear, the number of nerve fibres was averaged from the 10 habenular perforatae in each turn.

To examine the synaptic ribbons, the animal was decapitated and its cochlea was removed and quickly placed into cold PBS, and then perfused rapidly with 4% paraformaldehyde in PBS buffer followed by brief (10 min) post-fixation at 4°C. The cochlea was then transferred back into PBS and the bone over the middle-ear-facing portion of the cochlear spiral was removed with fine forceps. After removing the tectorial membrane, the cochlea was permeated with 1% Triton X-100 in PBS for 60 min, incubated for 60 min in 5% goat serum in PBS and then immunostained sequentially with primary antibody (mouse anti-CtBP2 from BD Biosciences at 1∶100; catalog #612044) for 24 h at 4°C and secondary antibody (goat anti mouse IgG at 1∶1000, Invitrigen A21131) for 2 h at room temperature, with three intervening PBS washes. All antibodies used were diluted in 1% goat serum in PBS. After immunostaining, the cochlea was then decalcified in 5% EDTA and the basilar membrane was dissected into five or six pieces, mounted on microscope slides and coverslipped.

Confocal images were acquired using a laser scanning confocal microscope (Zeiss LSM 510 META) with 543 nm lasers for excitation. Image stacks were ported to image-processing software (Lsmix and ImageJ), where the immunoreactive punctae of CtBP2 in the microscopic field were counted and divided by the total number of IHC nuclei. The number (presented as mean ± SEM) and position of these immunoreactive puncta were used to quantify IHC-SGC synaptic ribbon of every group.

### E. Statistical Analysis

All data values in the present paper are expressed as mean ± SEM and post-hoc multiple comparisions were performed using Tukey’s test following two-way ANOVA. SigmaStat for Windows was used for these statistical analyses. The significance level is indicated by the number of asterisks (*: p<0.05 and **: p<0.01, respectively).

## Results

### A. Changes in ABR Threshold

**Figure 2:**
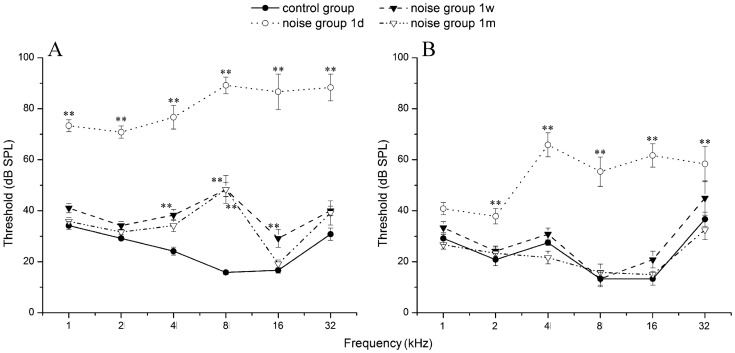
ABR thresholds shift and recovery after noise exposure at 110 dB SPL (A) and 105 dB SPL (B) for 2h. Thresholds shown are group means (± SEM) for 6 amimals tested per group. For simplicity, only data of control, 1dPE, 1wPE and 1mPE were shown. *: p<0.05, **: p<0.01.

In this study, hearing thresholds were evaluated by frequency specific ABR. Noise exposures at the two SPLs in the study caused an immediate, significant ABR threshold shift (TS) as shown in Figure 2 and Tables 1 and 2. The averaged threshold across the frequency range from 1 to 32 kHz increased from 25.14±3.42 and 23.47±1.71 dB SPL pre-exposure to 80.83±2.71 and 53.31±2.53 dB SPL 1d after 110 and 105 dB noise exposure respectively. The largest TS was seen at 8 kHz from 15.83±0.83 to 89.17±3.27 dB SPL for 110 dB noise exposure and at 16 kHz from 13.33±2.47 to 61.67±4.59 dB SPL for 105 dB SPL noise. Although the TS caused by 110 dB SPL noise didn’t recover even 6mPE at 4 and 8 kHz, there was no significant TS 1wPE after the exposure to 105 dB SPL noise (Table 2).

**Table 1:** ABR thresholds shift and recovery after noise exposure at 110 dB SPL for 2 h (mean ± SEM).

Group	1 kHz	2 kHz	4 kHz	8 kHz	16 kHz	32 kHz
Control	34.17±1.54	29.17±0.83	24.17±1.54	15.83±0.83	16.67±1.05	30.83±2.39
1dPE	73.33±2.39**	70.83±2.39**	76.67±4.65**	89.17±3.27**	86.67±6.91**	88.33±5.23**
1wPE	41.07±1.83	34.17±1.54	38.33±2.11**	48.33±2.79**	29.17±3.52**	40.00±1.83
1mPE	35.83±1.54	31.67±1.05	34.17±2.39	48.33±5.43**	19.17±1.54	39.17±4.73
6mPE	41.67±1.67	36.67±1.67	37.50±2.14**	45.00±7.74**	23.33±2.10	36.67±1.05

* :p<0.05,

** :p<0.01.

**Table 2:** ABR thresholds shift and recovery after noise exposure at 105 dB SPL for 2 h (mean ± SEM).

Group	1K	2K	4K	8K	16K	32K
Control	29.17±2.39	20.83±2.39	27.50±1.12	13.33±3.07	13.33±2.47	36.66±2.79
1dPE	40.83±2.39	37.83±3.06**	65.83±4.73**	55.33±5.73**	61.67±4.59**	58.33±6.91**
1wPE	33.33±2.47	24.17±2.01	30.83±2.39	13.33±2.47	20.83±3.27	45.00±6.71
2wPE	32.50±2.81	20.33±1.67	34.17±1.54	20.83±5.07	16.17±0.83	37.00±2.77
1mPE	26.67±1.67	23.33±2.47	21.67±2.47	15.83±3.27	15.00±1.29	32.50±3.82

* :p<0.05,

** :p<0.01.

A two-way repeated measure ANOVA was performed using time *re:* noise treatment as the main factor and the frequency as a co-variant on 6 guinea pigs to which ABR threshold measurement was repeated before and 1 day, 1 week, and 1 and 6 month(s) after the 110 dB SPL noise exposure (1dPE, 1wPE, 1mPE, 6mPE). A significant effect for the main factor (time re: noise exposure) was found (*F_4,179_* = 416.39, p<0.001). The thresholds after noise were compared with control thresholds at each frequency in post-hoc pairwise comparisons (Tukey tests). Significant changes are indicated by asterisk in Table 1. While significant shifts were evident across all frequencies at 1dPE. By 1mPE, significant difference was only found at 8 kHz. These data indicate that the 110 dB SPL noise exposure caused a permanent threshold shift (PTS) in the middle frequency region.

Similarly, a two-way repeated measures ANOVA was performed on ABR thresholds in 6 animals that were tested before, and at 4 different time points after the 105 dB noise (1dPE, 1wPE, 2wPE and 1mPE, Table 2). A significant effect for the time *re*: noise exposure was also found (*F_4,179_* = 86.00, p<0.001) in this group. Post-hoc pairwise comparisons showed that ABR thresholds were significantly elevated only at 1dPE, with the highest threshold shift (TS) at 16 kHz. At 1wPE, the thresholds for all frequencies were almost restored to their pre-exposure values and remained stable thereafter.

### B. Cochlear Output Changes in Compound Action Potential (CAP)

**Figure 3:**
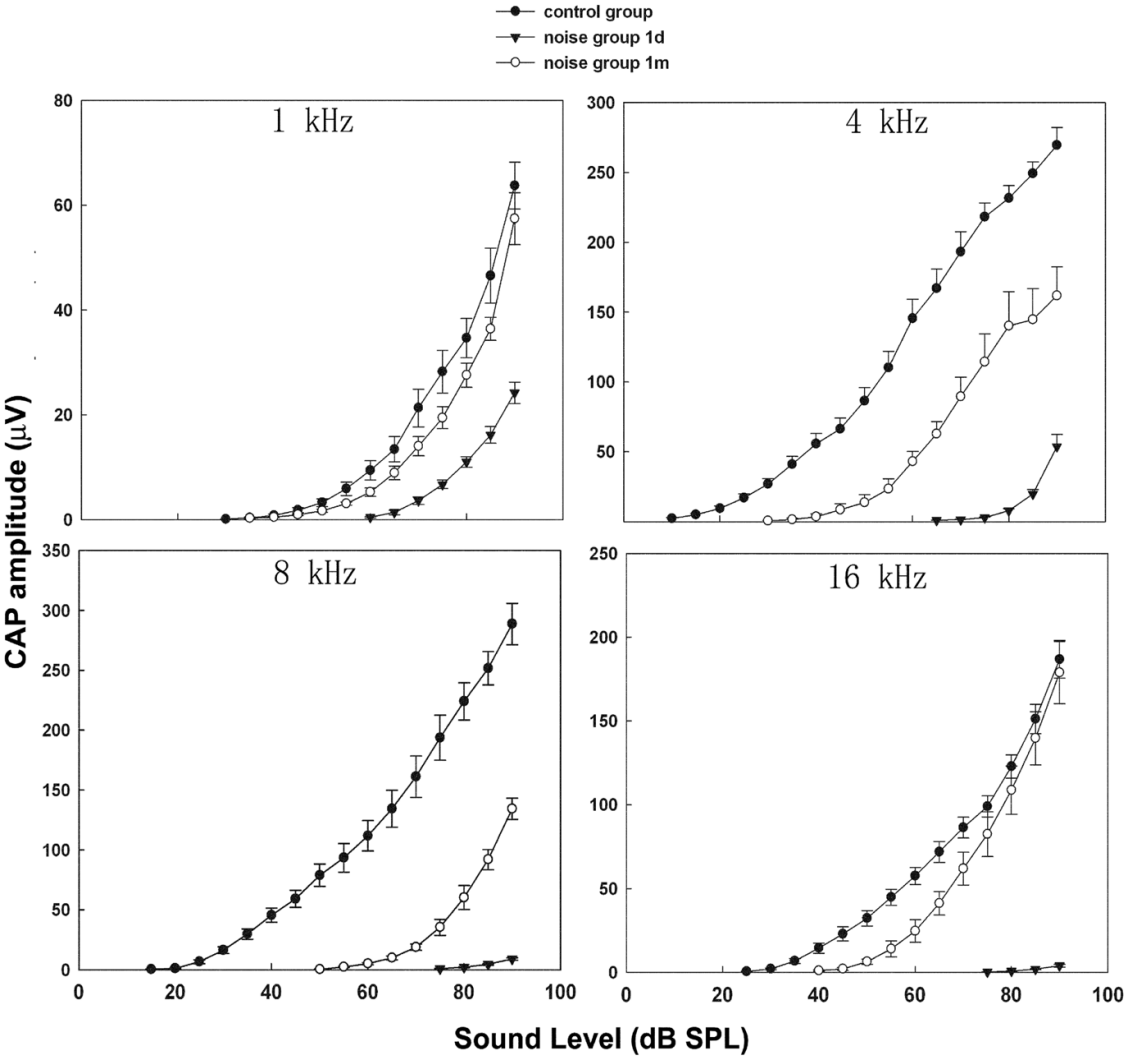
CAP I/O functions obtained from groups of no-noise control, 1dPE and 1mPE over 4 different frequencies (1, 4, 8 and 16 kHz) after 110 dB SPL noise exposure for 2 h. The vertical bars are SEMs.

To evaluate the impact of 110 dB SPL noise on CAP I/O functions, CAP was recorded from groups of no-noise control, 1dPE and 1mPE (n = 6 ears for each group, one from each animal). Figure 3 shows the I/O curves of CAP at four frequencies. It is clear that across the range of the tested frequencies and sound intensities, CAP amplitude was greatly depressed immediately after the noise exposure. The reduction in CAP amplitude is proportionally larger at the lower sound levels than at the higher ones, corresponding to the elevation in ABR thresholds at this time point. At 1mPE, the CAP I/O curve was largely recovered at 1 kHz across the whole range of sound levels and almost fully recovered at high sound levels but remain reduced at the lower sound levels at 16 kHz. At two other middle frequiencies where ABR threshold shift remained significant even at 1mPE, the I/O curves were only partially recovered across the intensity range.

The maximal CAP amplitude was measured and compared across these three groups in the middle frequency region where PTS was seen on ABR testing. In the control animals, the maximal CAP amplitude was on average 269.43±12.75 µV and 288.68±17.27 µV at 4 and 8 kHz respectively. In the noise-damaged ears 1 m after 110 dB SPL noise, the maximal CAP amplitudes were as low as 134.33±8.79 µV at 8 kHz, which is more than a 50% reduction from the control group (t = 7.034, df = 10, p<0.05) at this frequency. The amplitude at 4 kHz was also significantly reduced to 161.79±20.61 µV at this time point (t = 3.329, df = 10, p<0.05 ).

**Figure 4:**
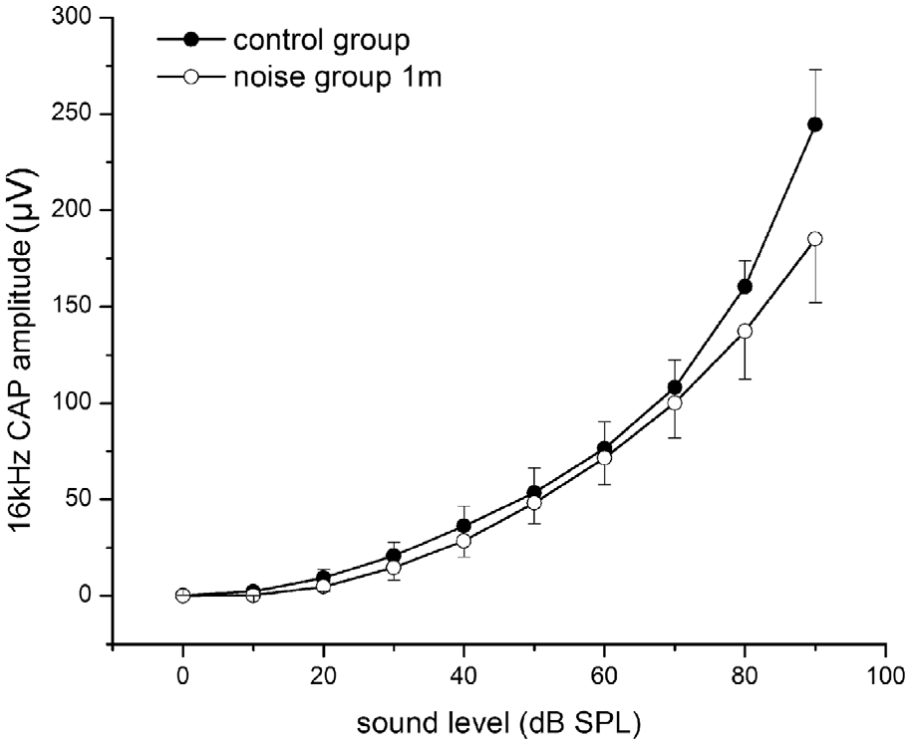
CAP I/O functions obtained at 16 kHz from no-noise control and 1mPE at 105 dB (n  = 6 and 5 respectively). The vertical bars are SEMs.

In animals exposed to 105 dB SPL noise, the I/O curve of CAP showed no threshold difference 1mPE as compared to the control group, in agreement with the temporary nature of the threshold shifts. A slight decrease in the maximal amplitude was seen at 16 kHz, as shown in Figure 4. The maximal amplitude at this frequency was reduced to 185.10±32.98 µV versus 244.48±28.61 µV in the controls, and did not recover. However, this difference was not statistically significant (t = 1.367, df = 9, p = 0.205).

### C. Damage of Afferent Innervation to IHCs

**Figure 5:**
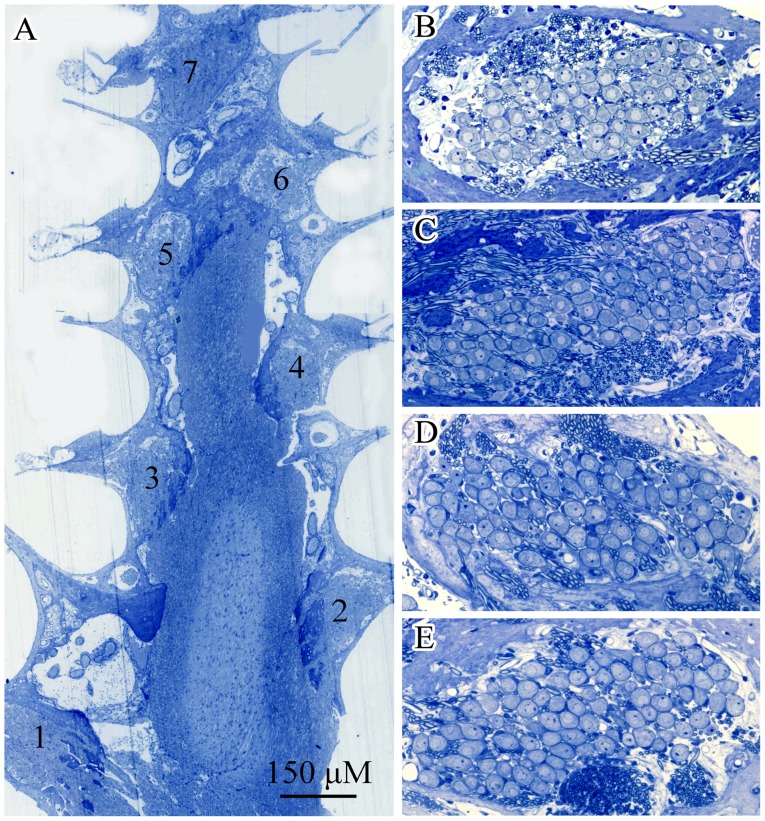
Images of cross-sections of modiolus. A: a whole view to show the 7 locations from basal to apical turns. B, C, D and E: representative views of SGNs in Rosenthal canals from different groups (B: control, C, D and E: 1dPE, 1mPE and 6mPE to 110 dB noise respectively). No obvious SGN loss was seen in the images obtained at different times after the noise exposure.

**Figure 6:**
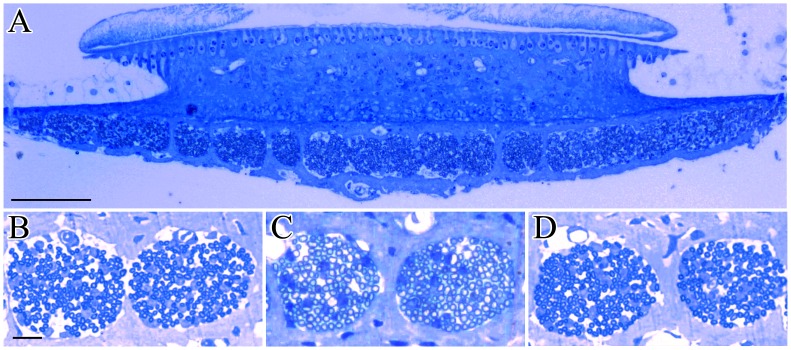
Cross sectional views of habenula perforata. A. Overall images of habenula perforata. B, C and D magnified habenula perforata of control, 1dPE and 1mPE to 110 dB noise samples. The ANF numbers were counted from the magnified images. Scale bar: 50 µM.

The brief noise exposure at the two levels failed to cause significant loss of hair cells (data not shown). The damage to the IHC afferent innervation caused by the brief 110 dB noise exposure was quantitatively evaluated by comparing the samples obtained at 1dPE, 1mPE and 6mPE with those of the control subjects. Specifically, we counted the number of SGNs in cross-sections of Rosenthal canal in the cochlear modiolus (Figure 5), as well as the ANFs in the osseous spiral lamina (Figure 6). The SGNs were counted at 7 locations (marked in Figure 5A) from basal to apical turns. At each location, the SGN count was calculated by averaging the numbers from 10 slices across a distance of 200 µm. The counts from locations 1 and 2, locations 3 and 4, and locations 5 and 6 were combined to make the values for the basal, 2^nd^ and 3^rd^ turns respectively (the SGN number for location 7 was used as the value for apical turn). To quantify the number of ANFs in each osseous spiral lamina sample, cross-sections of the cochleae parallel to the modiolus were obtained at the sites where the “holes” facing habenula perforata were well separated (as shown in Figure 6) in order to count the number of fibers in each hole. The number of ANFs at each hole was calculated and averaged over 10 holes for each turn.

In Table 3, the changes in SGN counts across groups are summarized. The SGN number was slightly smaller in samples obtained after noise exposure as compared with the control group. However, two-way ANOVA against the factors of time *re*: noise exposure and location (turns) failed to show any significant effect for the noise exposure on the SGN numbers for basal and second turn which are correspond to the frequency regions higher than 1 kHz (*F_4,83_* = 1.965, p = 0.109). The statistic power achieved in the test performed for time factor between the control and 6mPE was found to be 0.458 (with α  = 0.05, n  = 12). This moderate power makes it hard to infer that there is no effect of the noise on the SGN death.

**Table 3:** Comparison of SGN counts in Rosenthal canal at different locations across groups.

Group	Basal turn	Second turn	Third turn	Apical turn
ctrl (n = 12 ears)	87.13±2.43	73.72±1.23	66.35±1.05	87.12±2.17
1dPE (n = 6 ears)	86.23±0.80	73.33±0.79	67.77±0.97	83.23±0.97
1wPE (n = 6 ears)	83.73±1.24	72.37±1.78	66.83±1.41	80.61±3.79
1mPE (n = 6 ears)	78.12±5.34	71.58±5.17	61.98±3.19	79.00±6.07
6mPE(n = 12 ears)	81.83±1.23	71.68±0.95	63.29±1.05	82.35±1.18

Note: the 6 ears for 1dPE, 1wPE, and 1mPE are from 6 animals.

Table 4 shows the number of nerve fibers/perforata as measured from different time groups (re: noise exposure). Corresponding to the change of SGNs, the number was slightly smaller across turns at 1dPE, 1wPE and 1mPE compared with the control value. However, ANOVA for the factors of time *re*: noise exposure and location (turns) shows no significant effect of noise exposure on the ANF numbers for basal and second turn which are correspond to the frequency regions higher than 1 kHz (*F_3,59_* = 1.743, p  = 0.170).

**Table 4:** Comparison of ANF counts in habenula perforata at different locations across groups.

Group	Basal turn	Second turn	Third turn	Apical turn
Ctrl (n = 12 ears)	159.19±3.04	112.26±4.06	72.68±3.22	52.29±1.62
1dPE (n = 6 ears)	153.4±1.71	109.13±0.715	71.85±0.09	52.05±0.12
1wPE (n = 6 ears)	152.78±0.80	108.97±0.96	71.75±0.76	51.88±0.81
1mPE (n = 6 ears)	150.89±2.80	108.63±1.53	72.93±2.75	51.58±2.07

Note: the 6 ears for 1dPE, 1wPE, and 1mPE are from 6 animals.

### D. Changes in Ribbon Count

**Figure 7:**
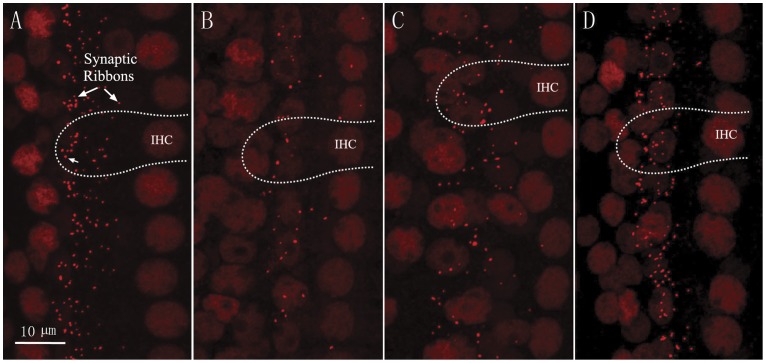
Confocal images of IHC area from different time groups. A: control, B, C and D: 1dPE, 1wPE and 1mPE to 105 dB noise respectively. Pre-synaptic ribbons were rendered visible as CtBP2-immunoreactive puncta, red. Dashed lines indicate the outlines of selected IHCs.

Immunostaining of the cochlea sensory epithelium for the CtBP2, a component of the pre-synaptic ribbon, rendered the IHC-SGN synaptic ribbons and the cell nuclei visible as shown in Figure 7. Confocal imaging was used in this study to quantify the change in synaptic ribbons after a 2 h noise exposure at 105 dB SPL. The numbers of ribbons were counted from apical to basal turns to establish ribbon-count cochleograms.

Figure 7 shows the representative ribbon images obtained from samples of different time groups around the 16 kHz region, where the greatest transient ABR threshold shift was seen. In IHCs of control ears, the overwhelming majority of the synaptic ribbons were located near the basal surface of the cell. In noise damaged ears obtained at 1dPE, only few remaining ribbons were seen, and the size of the residual ribbons appear to be larger and distributed upwards towards the nuclei of IHCs (Figure 7B). At 1wPE, the ribbon number was partially recovered but the change in the distribution of ribbons was still obvious. Further recovery in ribbon number was seen between 1wPE to 1mPE. This is accompanied by recovery in ribbon size and location.

**Figure 8:**
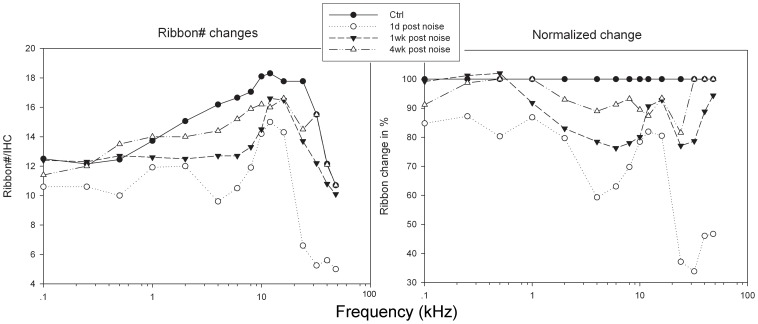
Graphs showing the changes in ribbon counts across the cochlear regions after 105 dB SPL noise exposure. 7A is actual changes in number, and 7B percentage change from control (i.e. maximal) values.

Figure 8 shows the changes in ribbon-count cochleogram (8A the absolute ribbon #/IHC across the whole cochlea, 8B percentage change in ribbon number) caused by the noise. Compared with the control group, ribbon counts across the whole cochlear region displayed a massive loss (on average ∼40%) at 1dPE with reductions of about 15–35% at low frequency regions and 60–70% at high frequency regions. The ribbon count at frequency regions lower than 1 kHz recovered completely at 1wPE and remained stable thereafter. The ribbon counts of middle and high frequency regions also recovered greatly at 1wPE. By 1mPE, the ribbon loss between 1 and 40 kHz was only roughly 10%.

### E. Changes in Temporal Processing Induced by Noise Which does not Cause PTS

**Figure 9:**
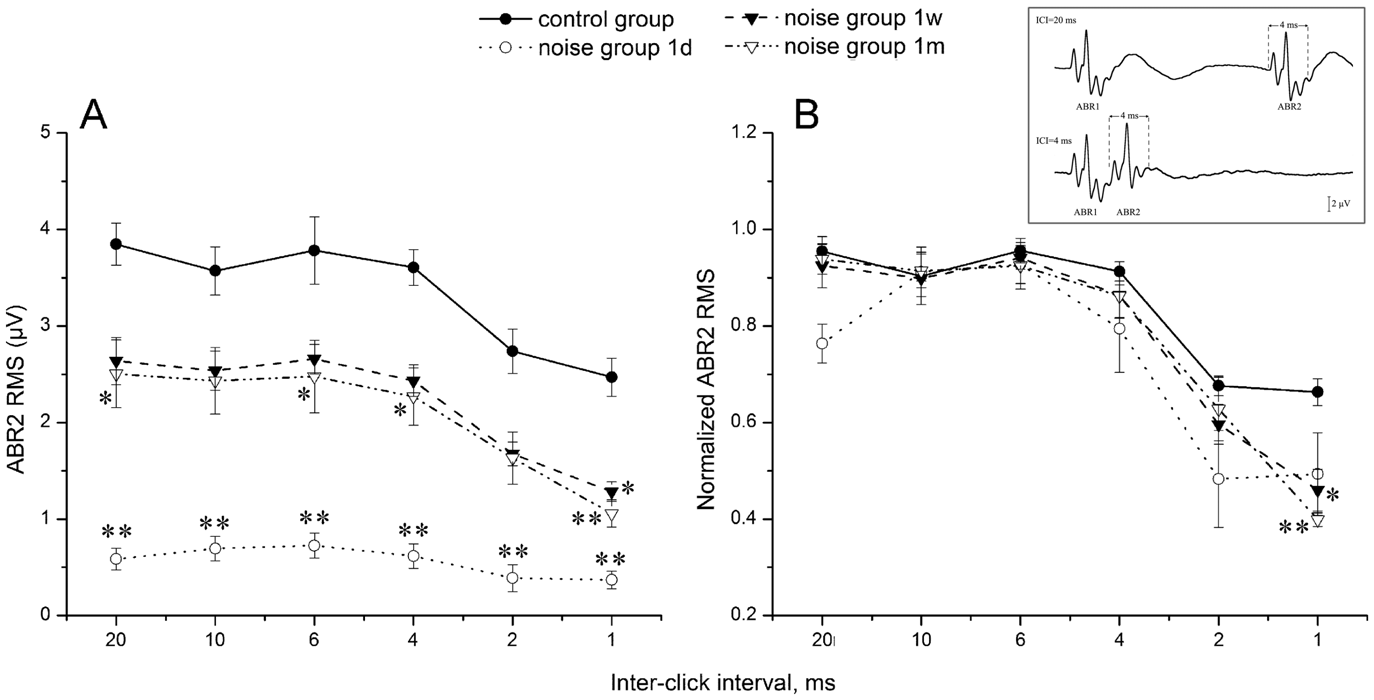
Graphs showing the changes of absolute ABR2 RMS (A) and normalized ABR2 RMS (B) with decreasing inter-click interval after 105 dB SPL noise exposure. For simplicity, only data of control, 1dPE, 1wPE and 1mPE were shown. *: p<0.05, **: p<0.01.

The impact of the noise exposure at 105 dB SPL which did not cause PTS was evaluated by the ABR responses to time-stress stimuli: paired clicks with varying ICIs. Our data analysis is focused on responses to clicks presented at a moderate sound level (70 dB peSPL). As shown in Figure 9A, the 105 dB SPL noise exposure caused an immediate and dramatic decrease on ABR2 amplitude at 1dPE. The ABR2 amplitude substantially recovered at 1wPE but remained significantly lower than the control value even at 1mPE. A two-way ANOVA for the main factors of time point *re*: noise exposure and ICI showed a significant effect for the noise exposure factor (F*_4, 179_* = 74.13, p<0.001). In addition, the ABR2 amplitude-ICI function was compared across the groups in Figure 9B, in which the ABR2 amplitude was normalized against the largest ABR2 obtained at a long ICI (usually 20 ms). In addition to the overal reduction in ABR amplitude, 9B shows that the ABR2 amplitude-ICI function reduces faster with ICI decrease at some PE time points than the control. To quantify the difference, a two-way ANOVA for the main factors of time point *re*: noise exposure and ICI was performed. Again, the result showed a significant effect for the noise exposure factor (F*_4, 179_* = 4.73, p = 0.001). The post-hoc pairwise tests (Tukey) showed that at 1 ms ICI, the normalized ABR2 amplitude was signficantly lower in the 1mPE group than in the control group (p = 0.002). Although the reduction in the normalized ABR2 at 1 ms ICI was already evident at 1dPE, the difference was not signficant (p  = 0.12). These data indicate that, in contrast to the recovery in hearing sensitivity after the noise, suprathreshold neural responses to the time-stress signals is deteriorated within the period of observation.

## Discussion

In the present study, the effect of noise exposure on afferent innervation to the cochlea of guinea pigs was explored at two noise levels, and 105 dB SPL was found to be the maximal noise level that did not cause PTS for a brief exposure of 2 h. This is similar to data reported previously [3]. The 110 dB noise depressed CAP amplitude substantially (Figure 3), while the 105 dB noise did not (Figure 4). No significant loss of SGNs was seen up to 6mPE to 110 dB noise. Corresponding to the CAP recovery after 105 dB noise exposure, the noise induced damage on ribbon synapses appears to be largely reversible in guinea pigs, unlike in the cochleae of mice [2]. However, in spite of a full recovery of auditory sensitivity and ribbon counts, the deterioration in auditory temporal processing extended to a long period. At this moment, we cannot simply attribute the deterioration to the damage on ribbon synapses. However, there is a possibility that the ribbon synapse damage is responsible, at least partially for the functional deterioration because the damage is the only finding of morphology damage resulted from this noise exposure.

Of note, there appears to be a clear cross-species difference between guinea pigs and mice with respect to noise-induced damage to type I afferent innervation in the cochlea. This cross-species variation should not be ignored when generalizing the phenomenon of noise induced SGN death to humans. The difference is demonstrated in several ways. Firstly, the ribbon loss in guinea pigs, as seen in this report, is much more reversible when compared to that previously reported in mice [2]. In the mouse study, the ribbon loss observed shortly after the noise exposure was roughly 60% and the recovery was ∼10%, leading to a 50% permanent loss, which was matched by the loss of SGNs two years later. In the present study, as well as that by Liberman’s group [3], the initial ribbon loss in guinea pigs was found to be comparable to that of mice. However, a much larger amount of recovery was seen within a month in the present report, which was not documented previously [3]. Secondly, no significant SGN loss was found 6mPE for 110 dB SPL noise in the present study. A recent study in guinea pigs [3] found a similar result: the SGN loss seen 2 years after a noise exposure of 105 dB SPL was less than 25% from a small sample of 3 ears. Thirdly, CAP amplitude was largely recovered in the present study after the 105 dB noise, suggesting a reconnection of the damaged IHC-SGN synapse. Since the slow degeneration of SGNs after noise-induced damage to IHC-SGN synapses is likely due to the loss of trophic support from IHCs and supporting cells, the reconnection between IHCs and SGNs would ensure the survival of SGNs. In light of the present findings, it is tempting to speculate that the decreased loss of SGNs over time in guinea pigs is due to reconnection of damaged synapses and there is a stronger self-repair at the synapses in the cochlea of guinea pigs as compared to mice. Finally, it is worth noting that in terms of hearing loss, the cochleae of mice are less sensitive than those of guinea pigs. For brief exposures about 2h, noise levels above 105 dB SPL (i.e. 110 dB) can cause PTS in guinea pigs, whereas levels above 115 dB SPL are required to cause PTS in mice, based upon our own experience. However, a massive SGN loss was seen after a noise exposure of 100 dB in mice, well below the maximal level which gives rise to no PTS in this species.

The massive SGN loss seen in mice naturally raises a serious issue about human noise safety standards which are established largely based upon the threshold shifts or sensitivity changes seen after different levels of noise exposure. If a similar SGN loss occurs in humans, it means that noise exposure that does not cause hearing loss may still severely damage the auditory system. However, the data in the present report highlight the need to study cross-species differences before generalizing to humans.

The mechanisms behind decreased SGN death in guinea pigs compared to mice are not clear. As discussed above, the SGNs are likely to die from loss of trophic support of IHCs and supporting cells after the disconnection between IHCs and SGNs caused by noise damage. The massive SGN death in mice after the noise exposure and the change in ribbon counts suggest that damaged synapses do not recover in mouse cochleae. However, many previous studies using guinea pigs and rats found that noise-induced damage to the ribbon synapse was largely reversible [5], [14]–[15]. Although this conclusion was criticized as not being quantitative and myopic with respect to potential long term consequences, and challenged by Kujawa and Lieberman based on their mice data [2], the present study clearly demonstrates a better repair ability in the cochleae of guinea pigs than that reported in mice. One may argue that the lower SGN loss seen in guinea pigs up to 2 years after noise exposure is due to the longer life span of guinea pigs and therefore possibly slower degeneration of SGNs after disconnection with IHCs. The fact that the rate of SGN loss following chemical or mechanical damage to hair cells also has been found to be highly species dependent has cited to support this argument [16]–[17]. Therefore, differing lifespans between guinea pigs and mice could be postulated to contribute to the different profiles of noise-related primary SGN degeneration across these two species. However, this possibility is unlikely because innervation is largely recovered, as indicated by the recovery of ribbon counts and the CAP amplitude. Since the IHC-SGN synapses are re-connected, there is no reason that these SGNs would die other than through natural aging.

The ribbon count has been challenged as an appropriate indicator of ribbon synapse connections. In a study using double staining against ribbon and a post-synaptic marker, glutamate receptor 2/3(GluR2/3), it was found that ribbon loss was less than that of GluR2/3 after noise exposure [3]. Therefore, the authors of that study suggest that ribbon count may underestimate the damage to synapses. This claim is questionable for several reasons. Firstly, labeling of GluR2/3 may not appropriately represent post-synaptic structures because this receptor is reduced or down-regulated when the synapse is exposed to glutamate [18]–[19], as a self-protection mechanism. Therefore, using other post-synaptic markers is suggested to verify the relationship between ribbon loss and the post-synaptic damage. Such a study is currently underway in our labs. Secondly, the SGN loss found 2 years after noise exposure in mice was matched by the reduction in ribbon counts in mouse cochleae [2]. This suggests that the ribbon count satisfactorily indicates the number of functional synapses under the assumption that the SGNs that lose synaptic connection with IHCs should have died due to the loss of trophic support. Thirdly, the CAP amplitude recovery was accompanied by ribbon count recovery in the present study, suggesting a functional reconnection of the ribbon synapses in the guinea pig cochlea.

In combination with previous studies in guinea pigs and chinchillas which have suggested that noise- or drug-damaged terminals either recover or regenerate [5], [14]–[15], we suggest another hypothesis for the discrepancy between guinea pigs and mice in the profile of ribbon changes as well as SGN degeneration following noise exposure: the intrinsic ability to self-repair following acoustic injury in the inner ear is different across species, and increased synaptic restoration leads to less delayed-onset SGN loss in guinea pigs than in mice. At this moment, we have no explanations for why the repair of the ribbon synapses is more effective in guinea pigs than in mice.

The ribbon synapse is characterized by the bar structure (the ribbon) in the presynaptic zone. The ribbon is considered a structure that facilitates the fast release and recycling of neurotransmitter [4]. Therefore, the ribbon structure is likely responsible for the high-resolution of temporal processing of the auditory system [4], [7]–[8], [12]. This is supported by the deterioration in temporal processing in mice with a mutation on Bassoon [20], a key protein that anchors the ribbon to the active zone of the presynaptic membrane. Since noise can significantly damage ribbons, it is likely that temporal processing ability is negatively affected by noise exposure. This is supported by the data obtained in the present study.

There are several potential mechanisms for the deterioration in temporal resolution after noise damage to ribbon synapses. First of all, temporal processing may be negatively affected as the result of a reduced number of functional channels. Each cochlear IHC is innervated by up to 30 individual type I SGNs. Temporal information can be coded by integrating the responses from multiple channels as suggested by volley theory [21]. The loss of functional auditory channels as a result of synaptic damage by noise could result in the reduction of the volley effect. Based upon our data, however, the contribution from this mechanism is likley small, because the time line for the changes in the number of functional channels is different than for changes in termporal processing; our study demonstrated that the deterioration in temporal processing is less at 1dPE, when the reduction of the functional channels was largest. Secondly, the ribbon synapses that initially survived noise exposure were functionally damaged. For the same reason mentioned above, this mechanism is also unlikely. Thirdly, repaired synapses are poor at temporal processing. Since more deterioration in temporal processing is seen 1 month than 1 day after the noise, we assume that the third mechanism is more important. It is of important and interesting to further investigate the mechanisms for the poor temporal resolution in repaired synapses and how long the deterioration continues.

It is clear that the noise-induced “silent” damage to the IHC-SGN synapse results in deterioration of temporal processing. This finding is important because low-level noise exposure exists around us in much of modern society. According to current safety standards, such noise exposure is considered safe. While each significant exposure may cause only subtle damage without a PTS, the effect can be cumulative. It is possible that such damage is related to the reduced temporal resolution observed in aging subjects [22], which is considered as the major problem in signal processing with aging and the reason for difficulty in speech perception [23]–[28]. Since the ribbon synapse in the cochlea is the first limiting structure for temporal processing in the auditory system, it is likely to be one of the loci responsible for this deterioration. It is possible that ribbon synapses in the cochlea could be damaged by cumulative exposure to noise and/or other hazardous factors sub-clinically, and that this is the main reason for the deteriorated temporal processing seen in aging subjects. This connection is supported by the concordance between the deterioration in temporal processing seen in aging subjects and the change in temporal processing after damage to the ribbon synapse based upon the functional roles of this structure and the data in the present study.
